# Laboratory systems as an antibacterial resistance containment tool in Africa

**DOI:** 10.4102/ajlm.v5i3.497

**Published:** 2016-10-31

**Authors:** Iruka N. Okeke

**Affiliations:** 1 Department of Pharmaceutical Microbiology, University of Ibadan, Ibadan, Oyo State, Nigeria

## Abstract

**Introduction:**

As crucial as clinical laboratories are to preventing, identifying and managing resistance problems, laboratory scientists are among the most overlooked stakeholders. This review outlines the contributions that diagnostic laboratory systems should make toward all five of the World Health Organization’s 2015 strategic objectives for antimicrobial resistance containment.

**Laboratory systems in resistance containment:**

Antimicrobial susceptibility testing and surveillance are central to antibacterial resistance management and control and need to be implemented more commonly and closer to sick patients. However, the scope of tests that promote judicious antimicrobial use extend beyond susceptibility testing. Laboratory tests for pathogens or their associated biomarkers confirm or rule out specific causes of signs and symptoms associated with infection. Laboratory systems also provide critical support to infection control programmes. All of these functions promote rational antimicrobial use and contain the spread of resistance. Routine laboratory data supports the development of vaccines and other technologies that could ease the pressure placed by antimicrobials. Laboratories are also a rich source of information for health professionals, policymakers and the general public about the urgency of the resistance problem and progress in containing it.

**Conclusion:**

Laboratory systems are integral to antimicrobial resistance containment and contributions from African laboratories to addressing resistance need to be enhanced.

## Introduction

Antimicrobial resistance is a grand global challenge that is reducing the success and increasing the cost of treating infections. As with many complex problems, there are many stakeholders in resistance – patients, prescribers and dispensers are often cited.^1^ However, much less visible but high on the list are those stakeholders that develop and deploy diagnostic tests. Diagnostic testing is key to resistance containment and may in fact be the most important tool for increasing appropriate access to antimicrobials while simultaneously containing resistance.^2,3^ When appropriate tests are judiciously used within the context of an effective laboratory system, they lower selective pressure for resistance by promoting targeted and rational use of antimicrobials. They also lower drug and healthcare costs and can identify treatment failure caused by resistance, thereby unveiling the nature and scope of resistance and preventing its spread.^4,5^

Laboratory systems and laboratory professionals are key to identifying, mapping, quantifying and communicating about resistance. Patients with resistant infections can only be effectively and efficiently managed with laboratory support. The definitive evidence that resistance is a problem, as well as the fine description of the problem, comes from laboratories.^6^ Most critically, attempts to treat infections or presumed infections without laboratory input drive resistance by increasing unwarranted antimicrobial use, which is a needless selective pressure for resistant strains. Infectious patients, treated without laboratory support, are also more likely to remain infectious for longer and therefore spread their diseases. Laboratory system strengthening for resistance containment is needed everywhere on the globe, but particularly in African countries, where infection is the leading cause of disease and death.

It is now universally acknowledged, within and outside the scientific community, that deliberate and forceful steps across different sectors must be taken to contain resistance. The recent World Health Organization (WHO) global action plan on antimicrobial resistance^7^ outlines five strategic objectives for resistance containment, as follows:

1. Improve awareness and understanding of antimicrobial resistance through effective communication, education and training.
2. Strengthen the knowledge and evidence base through surveillance and research.
3. Reduce the incidence of infection through effective sanitation, hygiene and infection prevention measures.
4. Optimise the use of antimicrobial medicines in human and animal health.
5. Develop the economic case for sustainable investment that takes account of the needs of all countries, and increase investment in new medicines, diagnostic tools, vaccines and other interventions.

Most readers of the plan will appreciate that clinical laboratories and laboratory professionals will lead the second objective of surveillance and research. However, even though their role toward goals are less prominent, as summarised in Table 1, laboratory systems are essential for meeting every one of these objectives, as discussed below.

**TABLE 1:** The role of the laboratory system in meeting the objectives of the World Health Organization global action plan on antimicrobial resistance.

Strategic objectives	Clinical functions	Public health functions
Improve awareness and understanding of antimicrobial resistance through effective communication, education and training	Adequate training on resistance for antimicrobial prescribers and other health professionals. Patient education to reduce demand for unnecessary antimicrobials and adherence to essential regimen.	Updated reports on resistance to Health ministries and policy makers. Inform media and all stakeholders on AMR issues. Communicate the threat that non-therapeutic use of antimicrobials poses for resistance.
Strengthen the knowledge and evidence base through surveillance and research	Identify aetiology of human and animal infections. Monitor efficacy of antibacterial treatment. Communicate susceptibility testing results to clinicians. Pilot and then implement new technologies that could increase the access and speed of testing or reduce its cost.	Support research to develop point-of-care assays for the rapid diagnostic of bacterial infections. Implement quality assurance for susceptibility testing. Develop strategies for AMR surveillance at the human-animal and the human-ecosystem interface. Develop and implement national AMR laboratory based-surveillance plans.
Reduce the incidence of infection through effective sanitation, hygiene and infection prevention measures	Support infection control by identifying and segregating patients infected by resistant pathogens. Permit source-tracking for infections.	Promote prompt effective antimicrobial therapy so that pathogens have fewer opportunities for transmission. Prevent epidemics through early outbreak identification and improved management and containment. Provide laboratory support for assessing risk in health systems, for water supplies, in agricultural settings and for food evaluations.
Optimise the use of antimicrobial medicines in human and animal health	Allow for broad-spectrum regimen to be replaced with narrow spectrum drugs, thereby reducing the risk of antibiotic-associated infections.	Promote the application of surveillance data to national and regional drug policy.
Develop the economic case for sustainable investment that takes account of the needs of all countries, and increase investment in new medicines, diagnostic tools, vaccines and other interventions	Reduce drug costs by allowing the cheapest effective drug to be selected rationally.	Allow the true cost of resistance to be computed and tracked. Provide economic evidence to support the replacement of antimicrobial drug use with vaccine and other preventive strategies.

AMR, antimicrobial resistance.

## Objective 1: Improve awareness and understanding

Dissemination of information about antimicrobial resistance is key to effecting the behavioural change necessary to contain resistance.^8^ Communications from laboratorians are easily evidence-based. Diagnostic laboratory professionals in general, and microbiologists in particular, are in an excellent position to disseminate information on antimicrobial resistance to health professionals and the general public. Resistance is a health problem but is also a microbiological phenomenon brought about by the genetic change and selective pressure from antibiotics. Resistant organisms circulate among humans but also in other domesticated and wild animal species, as well as in the environment. Microbiologists can and should highlight the mechanisms by which resistance evolves and spreads and how these connect to risk factors for resistant infections in both humans and animals. Some microbiologists have played a pivotal role in public engagement projects and educational initiatives to drive home the message that antimicrobials must be conserved.^8,9^ For example, the UK Microbiology Society focused one issue of its comic, ‘Marvellous Microbes’, on antimicrobial resistance.^10^ Microbiologists and other scientists need to do more to communicate the urgency of the problem as well as the pivotal contributions that laboratories make to contain it. This is particularly true in Africa, where much of the general public and a significant proportion of health professionals have very little awareness of what diagnostic laboratories actually do, or of the biological basis for antimicrobial resistance.

## Objective 2: Strengthen the knowledge and evidence base through surveillance and research

### Resistant strains are identified by antimicrobial susceptibility testing

Antimicrobial susceptibility testing by minimum inhibitory concentration (MIC) measurement or disc diffusion is the gold standard for identifying resistance.^11,12^ For bacteria, once a laboratory is capable of isolating and identifying pathogens from clinical specimens, very few additional resources are needed to obtain a susceptibility profile. MICs are measured in a broth dilution assay or on solid media by agar dilution.^13^ To obtain MICs, standardised test and control cultures are inoculated into or onto media containing different dilutions of antimicrobial agent. For diagnostic testing, doubling dilutions are the most commonly-used format, although any geometric or arithmetic progression can be used to broaden the test range or increase precision. The MIC is the minimum concentration preventing growth under test conditions. Media composition, incubation temperature, inoculum size and quality, antibiotic format and incubation time can all affect the MIC and must be standardised.

Disc tests are the most commonly-used methods of bacterial susceptibility testing worldwide. They are simple and reproducible tests that determine whether a bacterial isolate is sensitive or resistant to a given drug or drug class. Standard discs containing specific concentrations of each antibacterial are placed on a calibrated lawn of bacteria. After incubation, a clear zone of no growth appears if the concentration of antibiotic around the disc is greater than the MIC. As the concentration of antibiotic in the medium decreases with distance of diffusion through the agar from the disc, the level of resistance is inversely proportional to the diameter of the clear zone of inhibition. Diffusion kinetics of the antibacterial in the test medium are also determinants of zone size, therefore test standardisation is key. Earlier challenges with reproducibility have been overcome by standardising methodologies and interpretations and by using commercially-available media, discs and inoculum standardisation tools.^14,15^ Thus today, disc diffusion tests are straightforward protocols that are easy to quality assure. If diffusion from a calibrated strip containing graded concentration of antibiotic, rather than a circular disc, an MIC can be obtained by a diffusion protocol. MIC strips are a somewhat costly but cost-effective and simple way to obtain an MIC, particularly in laboratories that are set up for disc testing.

The resistance crisis makes it impossible to continue to argue that antibiotic susceptibility testing is superfluous, a point that was made in many venues, even by experts, as recently as a decade ago.^16,17^ However, a common misconception in some parts of Africa is that antibacterial susceptibility testing is a reference laboratory-level technique. Susceptibility testing is best *supported* from central facilities and requires external quality assurance, but susceptibility testing of aerobic bacteria is designed to be *performed* at routine microbiology laboratories with minimal bacteriology facilities.^18^ The closer the site for culture and susceptibility testing is to the patient and the provider, the greater the chance that it will impact care for that specific patient and affect empiric prescribing at the relevant facility. Disc diffusion tests in particular are robust, reproducible and capacity can easily be built.^19^ Disc diffusion tests can even be rigorously performed in field laboratories equipped with a portable autoclave and a small incubator. Compiling and disseminating facility-level data, in the process of providing prescribers with a valuable tool for resistance control, can also help to garner the support of clinicians for laboratory services. Simple software, such as WHONET,^20,21^ can be used at the facility level to aggregate and analyse susceptibility data and have the added advantage of allowing these data to be forwarded to and integrated with national, regional and global surveillance data. WHONET will run on the most basic of computers, independent of platform, and capacity to enter, retrieve and analyse data can be built with ease. The software is available free, can be used on- or off-line, and the WHO collaborating centre for antimicrobial resistance provides free technical support.

A ‘can do’ narrative is essential for extending access to antibacterial susceptibility testing in Africa. In decades past, the perception that actual ‘high-tech’ methods, such as flow cytometry for CD4+ counts and molecular tests for viral load, were highly specialised, caused reservations about appropriately deploying HIV-management plans, including antiretroviral therapy in Africa. With carefully thought-out programmes that built capacity in parallel with infrastructure, many more patients than was originally envisaged are today able to access these services at the point of care. In Western countries, laboratories below the level of those that offer diagnostic support for highly-active antiretroviral therapy provide routine susceptibility testing, and the same is true historically of many African facilities that no longer offer testing today. Similar stories have unfolded around multidrug tuberculosis and malaria, for which the impracticability and long duration of testing has led to the development of rapid molecular Xpert^®^ tests and rapid diagnostic tests, respectively, providing many patients today with rapid diagnosis at the point of care.^22,23^ For HIV, tuberculosis and malaria, new technologies had to be developed and deployed to provide testing options in Africa. Antibacterial susceptibility testing, the much lesser challenge, based on much older methodology, has been less successfully rolled out to African patients and its neglect has important consequences for the delivery of care and resistance containment.^4^

Antibacterial susceptibility testing uses basic techniques that can and should be offered by a secondary or even primary care level laboratory with minimal microbiology resources. They are methods that any medical laboratory scientist with microbiology training can perform. The required equipment is small, robust and inexpensive and the costs of consumables and quality assurance are not high.

### Determining the aetiology of infections contains resistance

Bacterial infections can be life-threatening and while antibiotic use has a huge societal cost, most antibacterials are devoid of serious side effects. For these reasons, a significant number of people receive an antimicrobial prescription ‘just in case’. As soon as it can be shown that a patient’s signs and symptoms do not emanate from a bacterial infection, antibiotics can be withheld with confidence. Spot tests for viral causes of childhood diarrhoea and acute upper respiratory tract infection reduce unnecessary empiric use in those conditions. Simple point-of-care Streptococcal (Strep) throat swab tests have reduced antibiotic use in many parts of the world, because antibiotics are only ordered if the test is positive.^24,25^ For tests available in rapid point-of-care formats, results are available before initial therapy is begun and therefore unnecessary causes of empiric therapy can be prevented. In veterinary infections, point-of-care tests that delineate diarrhoea of bacterial aetiology from viral infections can be used on a farm to make an informed decision on the necessity for antibiotics.^4^

Rapid point-of-care tests have not been as effective as they might be because patients and their providers often disregard the results and administer antibiotics when these tests suggest that they are not necessary.^26^ Point-of-care testing may also be viewed as burdensome and even undermine care when it uncovers the fact that available therapies may not be effective choices.^27^ Nonetheless, testing and surveillance are essential for revealing inadequacies to policymakers as well as informing available options. Point-of-care tests, in particular, are exceptionally valuable in settings where the patient cannot easily return for follow-up, as is typical for many African health systems.^4,28^ Laboratory professionals have a key role to play in educating stakeholders and building their confidence in tests that optimise patient treatment and conserve antimicrobials. Going forward, development and deployment of multiplex tests that could help detect or rule out multiple causes of fever, diarrhoea or respiratory distress could further help to optimise antimicrobial use.

### Biomarker tests can rapidly narrow differential diagnoses

Culture and susceptibility testing remain the only reliable and affordable option for many clinics. The challenge of slow turn-around time will soon become a problem of the past. Rapid molecular and mass-spectrometry tests have now been developed and are being pre-tested and deployed in clinical laboratories that do not face severe resource limitations.^29,30,31^ While efforts are underway to increase the sensitivity and specificity of these tests and broaden their access, rapid aetiologic agent tests are only available for a few pathogens and in some cases are too expensive to be used routinely today in African settings. Much-needed multiplex tests are non-existent or rare. This is a pressing need that many African researchers are engaged in addressing but to which much more research activity needs to be devoted. In severe infections of likely bacterial aetiology, antibacterial chemotherapy must be instituted swiftly to avoid unnecessary mortality or death before susceptibility testing results are obtained. For this reason, biomarker tests that provide rapid results and can reliably identify a bacterial infection, then antibacterial therapy is only prescribed when a bacterial infection is present. C-reactive protein and procalcitonin are among useful markers of systemic bacterial infection that are not used, or are underused in Africa.^32,33,34^ In some cases, the sensitivities, specificities and positive and negative predictive values in African settings have yet to be evaluated making it difficult to advocate for their use.^34^ Similarly, markers of invasive diarrhoea, such as lactoferrin,^35,36^ could potentially help restrict excessive use of antibiotics in viral and other self-resolving gastroenteritis and need to be assessed and deployed in African settings.

Surveillance of resistance is critical for qualifying and quantifying the problem, and for informing essential empiric prescribing of medication. To a large extent, the precise magnitude of resistance is not known. However, we know there is a problem and most of what we know about its scope and volume comes from laboratory testing and surveillance, both core functions of a laboratory system.^37^ In Europe, for example, where surveillance is routine and has good coverage, there are good estimates of resistance and surveillance data have been used to prevent resistance rises or even bring resistance rates down.^38,39^ By contrast, where systematic surveillance is absent or rare, it is not clear what the major problems are, how big the problems are, or how best to tackle them. Surveillance data are least available from Africa^40^ and as such, the resistance problem is largely sketched from research data from small studies and clinical case reports. Those data sources indicate a worrisomely large and growing problem, the cost of which is difficult to estimate. As of 2015, with the absence of surveillance, only a handful of African countries had demonstrated little policy activity or evidenced interest in addressing resistance.^40,41^ In countries where resistance is at the forefront of policy-makers’ priorities, some kind of surveillance information is available.

One justification for swiftly and decisively applying appropriate alternate diagnostic tools is the long time that culture and susceptibility testing takes. Severely-ill patients must be treated empirically, so is the cost of tests that will yield results justified? As resistance becomes increasingly common, the justification increases. Surveillance data, if available, becomes less predictive when new resistances emerge. And when initial empiric therapy fails, the only fail-safe method to suggest an alternate chemotherapeutic course is to use laboratory testing information. The time to test results by conventional methods is long but, in most cases, it is well within the ideal time to make the necessary switch that could save a patient’s life.

### Surveillance and laboratory testing are critical

Surveillance and laboratory testing are critical to implementing alternatives to antibiotics that are technological solutions to the resistance problem. Surveillance determines the burden of a given infectious disease as well as the predominant pathogenic subtypes of its aetiological agent. Only with this information can vaccines be developed and deployed. Access to antimicrobial susceptibility testing has to increase, as do ways to integrate representative samples of infection testing into surveillance networks. Only with these developments will susceptibility information be optimally exploited in patient care and antimicrobial conservation. As we work to expand the reach of good testing and build fine surveillance networks, complementary technologies could be brought to bear on this problem. Difficulties with urban traffic and poor rural roads in warm moist tropical countries hamper the use of sentinel laboratories that could collect and process specimens from vast areas. Improving specimen and data transport systems, for example through application of aerial drones to convey specimens (building on an idea from Moses Bangura http://falling-walls.com/lab/news/Next+Einstein+Forum+sends+African+innovator+to+Falling+Walls+Lab+Finale+2016-7409) and mobile phones to return data could change the present situation in which susceptibility data are rarely available from poor countries and rural areas.

## Objective 3: Reduce the incidence of infection

### Laboratory systems are central to infection control

Antimicrobial resistant infections are increasingly hospital acquired. Laboratories can confirm outbreaks and are key to determining what happened and why. Importantly, the data they provide help to prevent the spread of infections within health facilities so that fewer individuals require curative, typically antibiotic-based interventions. Veterinary microbiology laboratories can help contain agricultural outbreaks and prevent infected meat products entering the human food chain.

### Surveillance allows for better burden estimates

Laboratory-based surveillance makes important contributions to information that is essential for disease control policy decisions. It was malaria surveillance data that were used to model the impact of antimalarial interventions and which ultimately revealed that the most valuable antimalarial intervention has been insecticide treated bed nets. There is thus rigorous evidence supporting a non-drug preventive strategy with significant impact on one of Africa’s most burdensome diseases. Models based on resistance surveillance could provide more insight about bacterial infections and the trajectory for drug resistance and thereby inform disease and resistance control.

### Laboratory systems as justification for development and deployment of disease control tools

For *Streptococcus pneumoniae*, decades of laboratory-based surveillance demonstrated that only a handful of serotypes accounted for most of the disease *and* most of the antibacterial resistance. These data aided the development of conjugate vaccine cocktails that are preventing resistant *S. pneumoniae* infections, an important cause of childhood illness and death in Africa, and continue to drive vaccine policy worldwide.^42,43^

The application of susceptibility information from laboratories to vaccinology is by no means limited to pneumococci. Until recently, oral antibacterials were the key tool used to control cholera outbreak size. When resistance became commonplace, not only were antibiotics less effective, but use of these drugs in outbreaks increased selective pressure for resistance. It is now acknowledged that while they cost more per dose than most antibacterial medicines, vaccines are the more cost-effective means for preventing pathogen spread in an outbreak and protecting the un-infected from the disease.^44^ Vaccines are so effective in this regard that scientists were able to justify building a stockpile of vaccines for use during outbreaks.^45^ A vaccine-based approach to outbreak control is particularly valuable in Africa, where the same geographic areas have seen repeated outbreaks in recent years.^46^ It is imperative that drawings are only made from the stockpile in the event of an outbreak that is verifiably cholera. Therefore, countries can request vaccines from this life-saving and resistance-averting stockpile only when aetiologic confirmation of cholera is obtained after laboratory testing.^47^ What this means is that laboratory systems will improve vaccine access.

Availability of CD4+ counting and viral load testing to Africans living with HIV has been crucial for the appropriate staging of infections and commencement of antiretroviral therapy. Testing will also help to identify antiretroviral resistance promptly, limiting its spread. Assuring effective antiretroviral therapy also prevents the dissemination of particularly problematic clones of opportunistic pathogens, epidemics of which are a matter of concern in Africa.^48,49^ An additional, often unstated benefit of laboratory-supported highly-active antiretroviral therapy programmes is that they relieve pressure from trimethoprim-sulphamethoxazole and other antibacterials that were used to prevent opportunistic infections in AIDS. Prophylactic antibacterials were the main tool used to prolong lives on the continent prior to the 2000s and were most probably selected for resistant bacterial clones and elements that have since narrowed antimicrobial options.^50,51^

## Objective 4: Optimise the use of antimicrobial medicines

### Informed prescribing for life-threatening invasive disease

For some life-threatening human infections, antibacterials must be prescribed as soon as possible after patient presentation, before the results of antibacterial susceptibility testing are available. Cases in point are bacteraemia and meningitis. In these infections, the patient is best served when recent local susceptibility information is available since this is the best way to guide empiric prescribing. When appropriate data are not available, more expensive reserve drugs are overused and/or treatment failures are more common. Even when such data are available, blood or cerebrospinal fluid cultures should still be performed for two key reasons. Firstly, patient-specific culture and susceptibility information allows caregivers to switch to a more appropriate antibacterial than the initial empiric choice. Such changes are most likely to be implemented for in-patients and are most valuable in resource-limited settings. Secondly, performing culture and susceptibility testing, even when empiric treatment is instituted, also accumulates vital information for treating other patients.

Blood and cerebrospinal fluid culture-derived susceptibility data are, for these reasons, among the most valuable types of susceptibility information for clinicians. Indeed, where resource limitations preclude culture and susceptibility testing for all specimens, prioritising blood and cerebrospinal fluid is the most cost-effective alternative.^51,52^ This is true even though blood culture is more tedious and more expensive than urine or stool culture, and more prone to invalid results. High-quality, life-saving blood culture information can be obtained even if automated blood culture machines are not available.^53^ Blood culture data can also be disseminated in geographic-specific ways to resistance-data networks (for example, ResistanceMap; http://resistancemap.cddep.org/). Sadly, many more physicians practising in Africa have access to culture and susceptibility testing for non-emergency samples than for blood.

### National and international drug policy

The availability of surveillance data can influence drug-use policy on national and even international levels. In Ghana, antibacterial resistance patterns of *Vibrio cholerae* isolates were the basis for altering national treatment guidelines and resistance reports from other areas to support the idea that vaccines, rather than antibacterials, should be the mainstay for cholera outbreak control.^54^ Similarly, archival resistance data from Nigeria refutes the hypothesis that chloroquine may select for fluoroquinolone-resistant bacteria, making it unnecessary to factor in bacterial resistance in future policy decisions about this antiplasmodial drug.^55^

Drug policies that are relevant to resistance include how different agents are used as well as which drugs are selected. More evidence is needed to determine whether antibacterial cycling or combinations might curtail resistance, and to guide drug use in veterinary settings to minimise the threat of resistance evolving in animal as well as human pathogens.

### Novel antimicrobial strategies

Evolutionary theory and emerging data suggest that treatments that diminish or clear infections without actually killing bacteria or inhibiting growth may have a lower propensity to be overcome by resistance. Thus, when preclinical candidates that inactivate bacterial toxins or prevent microbial adherence become available, they could have a significant impact on resistance containment.^52,56,57^ Narrow-spectrum drugs, peptides or even bacteriophages also offer promise because the selective pressure they exert does not extend to a significant proportion of the normal flora. Similar to vaccines and disease prevention tools, antivirulence, bacteriophage and other non-antibiotic therapies rely on precise identification of the aetiologic agent in every infection, before appropriate treatment can be selected and administered.

## Objective 5: Develop the economic case for sustainable investment

Antimicrobial resistance is a ‘tragedy of the commons’: a destructive paradigm created by a cheap, effective and widely-available resource (the antimicrobial drugs themselves), overuse of which incurs few costs for individuals but profound costs to society.^58,59^ A first step in preventing a tragedy of the commons is achieving a recognition of necessity. For antimicrobial resistance, the essential evidence for recognition comes from laboratories.^37^ With this evidence, it is possible to compute the losses from drug resistance as well as what might be saved if antimicrobials are conserved.^3,60,61^

Less extensive economic information is available from African countries but the economic justification for investment in resistance containment, in general, and laboratory contributions, in particular, is illustrated by a case of prolonged febrile illness in a Nigerian child with a history of empiric treatment with amoxicillin, fluoroquinolones and artesunate.^62^ When the child did not recover and was referred, blood culture revealed the aetiologic agent in the infection to be an extended-spectrum beta-lactamase-producing *Klebsiella pneumoniae* strain that was susceptible to imipenem, which cleared the infection. During her six-week illness, chemotherapy and supportive care cost almost $600.00. Only $387 was for the intravenous imipenem/cilastatin that eventually cleared the infection. Diagnostic testing, including microbiological tests, blood chemistry, radiology that supported this patient and uncovered the extended-spectrum beta-lactamase -producing aetiological agent cost $90.00.^62^

Challenges with antimicrobial access are as important to dealing with resistance as those produced by ‘excess’ in antimicrobial use. Far too many African patients do not have access to antimicrobials at all and/or the best option for treating their infections and minimising the threat of resistance.^63^ For those that do have access to some antimicrobials, surveillance data will indicate whether the access is to effective drugs – an important means for containing resistance.

Among the justifications given for inadequate application of laboratory systems for infectious disease management and resistance control are the cost of testing. However, as the Aboderin et al.^62^ case demonstrates, multiple empiric courses of antibacterial chemotherapy are costly, and supportive management of patients that are sickened by prolonged infections is even more expensive. Most importantly, in cases such as that, the correct course of therapy, when it is itself expensive, cannot be initiated without diagnostic support. In addition to the high monetary cost of diagnostic insufficiency of these practices, significant disability-adjusted life years are lost when clinicians attempt to manage infections without the necessary information.

## Ways forward

Without containing antimicrobial resistance, the Sustainable Development Goals and the Global Health Security Agenda cannot be met.^64^ This paper illustrates how laboratories are central to resistance containment and especially focuses on the need for laboratory system strengthening on the African continent where, if nothing changes, 4.15 million people are predicted to be at risk of dying from resistant infections annually by 2050.^65^ Healthcare providers need to make better use of existing laboratory services and demand necessary improvements. Robust laboratory systems will actually reduce medicine and care expenses, therefore the perceived high cost of laboratory services should not be a balance to better building and integrating laboratories into the health system.

African laboratories need to be better resourced so that they can deliver susceptibility information. This includes equipment, consumable resources and their supply chains, as well as development of the human resources to perform tests, store, curate and disseminate data, and apply these data to human and veterinary medicine and public health. Recent audits indicate that these resources are lacking in many African health systems, as is access to both internal and external quality assurance.^4,66^ Importantly, these are well-recognised needs, with well-known modalities for delivering them. Therefore, these shortfalls can and should be addressed. The data, and their application, also need to be worked into functional surveillance systems. Data from African countries are few and difficult to get, such that programmes that agglomerate data have described localities within the continent as being ‘data deserts’.^67^ Oases must be built through national surveillance programmes, something that could potentially be done through the WHO’s Global Antimicrobial Resistance Surveillance System.^68^

Expansion of access to currently-available susceptibility testing methods and techniques is needed, as well as better agglomeration and curation of existing data. For example, Gandra, Merchant and Laxminarayan point out that in low- and middle-income countries, where public-sector laboratories may not be able to perform testing, private-sector laboratories often provide this service. However, data from those laboratories, while useable for patient care, is often inaccessible for public health purposes.^67^ Where public sector data are available but largely serve lower-income patients than the private sector, careful laboratory-based surveillance could in fact be a useful pointer to differences in susceptibility rates among patients in different social strata.^69^ Connecting susceptibility testing data to patient care and public health is a weak point in many laboratory systems that *do* provide susceptibility testing. Moving away from notebooks that make data retrieval and agglomeration difficult may be one way to improve connections between susceptibility data generators and other healthcare providers. When electronic systems are introduced, resource-limited health facilities will be better served by free software systems with fewer capabilities than more complex systems requiring expensive subscriptions and difficult-to-access technical support.

In the not-too-distant future, innovation around laboratory testing for resistance and surveillance promises faster diagnostic testing and more responsive surveillance Next generation sequencing is increasingly affordable and can produce aetiologic information and extensive susceptibility profiles in a fraction of the time that is currently used to obtain more limited information by culture and susceptibility.^70^ Cost, the major barrier to sequencing, is rapidly dropping, and improved tools that allow scientists without bioinformatics skills to use genome information are being developed. In a few years, genome sequencing should be the method of choice for life-threatening infections, and outbreaks in particular. Similarly, it has been demonstrated that the major capital investments necessary to implement multiplex PCR tests or matrix-assisted laser desorption ionisation time-of-flight in clinical laboratory settings in North America can be offset by savings in medicines and hospital care.^71^ The scope and performance of point-of-care tests is improving and advances in nanoscience and microfluidics promise cheaper tests here as well.^33^ Better links between African research and clinical laboratories may help move these newer technologies into patient care.

### Conclusion

The limited information available suggests that those resistance-containment interventions that draw heavily from laboratory systems – such as infection control and surveillance programmes – when properly implemented, comprise the greatest evidence base for effectiveness. African health systems have a greater proportion of patients with infectious diseases, a greater incentive than others to conserve antimicrobials and therefore the greatest need to prioritise laboratory resources for antimicrobial resistance containment. African laboratories offering diagnostic testing and participating in resistance surveillance will need to be early adopters of some of these technologies, which will ultimately further optimise antimicrobial use and contain costly resistance.

## References

[1] Laxminarayan R, Chaudhury RR Antibiotic resistance in India: drivers and opportunities for action. PLoS Med. 2016;13(3):e1001974 http://dx.doi.org/10.1371/journal.pmed.1001974 26934098 10.1371/journal.pmed.1001974 PMC4775002

[2] Okeke IN, Peeling RW, Goossens H, et al. Diagnostics as essential tools for containing antibacterial resistance. Drug Resist Updat. 2011;14(2):95–106. http://dx.doi.org/10.1016/j.drup.2011.02.002 21398170 10.1016/j.drup.2011.02.002

[3] Mendelson M, Røttingen J-A, Gopinathan U, et al. Maximising access to achieve appropriate human antimicrobial use in low-income and middle-income countries. Lancet. 2015;387(10014):188–198. http://dx.doi.org/10.1016/S0140-6736(15)00547-4 26603919 10.1016/S0140-6736(15)00547-4

[4] Okeke IN Divining without seeds: the case for strengthening laboratory medicine in Africa. Ithaca, NY: ILR/Cornell University Press; 2011.

[5] Petti CA, Polage CR, Quinn TC, et al. Laboratory medicine in Africa: a barrier to effective health care. Clin Infect Dis. 2006;42(3):377–382. http://dx.doi.org/10.1086/499363 16392084 10.1086/499363

[6] Grundmann H, Aanensen DM, Van Den Wijngaard CC, et al. Geographic distribution of Staphylococcus aureus causing invasive infections in Europe: a molecular-epidemiological analysis. PLoS Med. 2010;7(1):e1000215 http://dx.doi.org/10.1371/journal.pmed.1000215 20084094 10.1371/journal.pmed.1000215 PMC2796391

[7] World Health Organization Global action plan on antimicrobial resistance. Geneva, Switzerland: WHO; 2015. 10.7196/samj.9644 26242647

[8] Castro-Sánchez E, Chang PWS, Vila-Candel R, et al Health literacy and infectious diseases: why does it matter? Int J Infect Dis. 2016;43:103–110. http://dx.doi.org/10.1016/j.ijid.2015.12.019 26751238 10.1016/j.ijid.2015.12.019

[9] Earnshaw S, Monnet DL, Duncan B, et al European Antibiotic Awareness Day, 2008 – the first Europe-wide public information campaign on prudent antibiotic use: methods and survey of activities in participating countries. Euro Surveill. 2009;14(30):19280. 19643056 10.2807/ese.14.30.19280-en

[10] Microbiology Online Marvellous microbes. 4th ed [page on the Internet]. c2016 [cited 2016 Oct 09]. Available from: http://www.microbiologyonline.org.uk/teachers/resources

[11] Jorgensen JH, Turnidge JD Susceptibility test methods: dilution and disk diffusion methods. In Jorgensen J, Pfaller M, Carroll K, et al., editors. Manual of Clinical Microbiology, 11th ed Washington, DC: ASM Press, 2015; pp. 1253–1273. http://dx.doi.org/10.1128/9781555817381.ch71

[12] Perilla M, Ajello G, Bopp C, et al. Manual for the laboratory identification and antimicrobial susceptibility testing of bacterial pathogens of public health importance in the developing world; WHO/CDS/CSR/RMD/2003.6. Geneva, Switzerland: WHO CDS Information Resource Centre; 2003.

[13] Wiegand I, Hilpert K, Hancock REW Agar and broth dilution methods to determine the minimal inhibitory concentration (MIC) of antimicrobial substances. Nat Protocols. 2008;3(2):163–175. http://dx.doi.org/10.1038/nprot.2007.521 18274517 10.1038/nprot.2007.521

[14] Ericsson HM, Sherris JC Antibiotic sensitivity testing. Report of an international collaborative study. Acta Pathol Microbiol Scand [B] Microbiol Immunol. 1971;217:Suppl 217:1+. 4325956

[15] Gradmann C Sensitive matters: the World Health Organisation and antibiotic resistance testing, 1945–1975. Soc Hist Med. 2013;26(3):555–574. http://dx.doi.org/10.1093/shm/hkt018

[16] Mabey D, Peeling RW, Ustianowski A, et al. Diagnostics for the developing world. Nat Rev Microbiol. 2004;2(3):231–240. http://dx.doi.org/10.1038/nrmicro841 15083158 10.1038/nrmicro841

[17] Polage CR, Bedu-Addo G, Owusu-Ofori A, et al. Laboratory use in Ghana: physician perception and practice. Am J Trop Med Hyg. 2006;75(3):526–531. 16968935

[18] World Health Organization Manual of basic techniques for a health laboratory. 2nd ed Geneva, Switzerland: WHO; 2003.

[19] Opintan JA, Newman MJ, Arhin RE, et al. Laboratory-based nationwide surveillance of antimicrobial resistance in Ghana. Infect Drug Resist. 2015;8:379–389. http://dx.doi.org/10.2147/IDR.S88725 26604806 10.2147/IDR.S88725 PMC4655947

[20] O’Brien TF, Stelling JM WHONET: an information system for monitoring antimicrobial resistance. Emerg Infect Dis. 1995;1(2):66 http://dx.doi.org/10.3201/eid0102.952009 10.3201/eid0102.950209 PMC2626837 8903165

[21] O’Brien TF, Stelling JM WHONET: Removing obstacles to the full use of information about antibiotic resistance. Diagn Microbiol Infect Dis. 1996;25(4):163–168. 8937840

[22] Thiam S, Thior M, Faye B, et al. Major reduction in anti-malarial drug consumption in Senegal after nation-wide introduction of malaria rapid diagnostic tests. PLoS One. 2011;6(4):e18419 http://dx.doi.org/10.1371/journal.pone.0018419 21494674 10.1371/journal.pone.0018419 PMC3071817

[23] Boehme CC, Nabeta P, Hillemann D, et al. Rapid molecular detection of tuberculosis and rifampin resistance. N Engl J Med. 2010;363:1005–1015. http://dx.doi.org/10.1056/NEJMoa0907847 20825313 10.1056/NEJMoa0907847 PMC2947799

[24] Ayanruoh S, Waseem M, Quee F, et al. Impact of Rapid Streptococcal Test on Antibiotic Use in a Pediatric Emergency Department. Pediatr Emerg Care. 2009;25(11):748–750. http://dx.doi.org/10.1097/PEC.0b013e3181bec88c 19864964 10.1097/PEC.0b013e3181bec88c

[25] Maltezou HC, Tsagris V, Antoniadou A, et al. Evaluation of a rapid antigen detection test in the diagnosis of streptococcal pharyngitis in children and its impact on antibiotic prescription. J Antimicrob Chemother. 2008;62(6):1407–1412. http://dx.doi.org/10.1093/jac/dkn376 18786938 10.1093/jac/dkn376

[26] Lubell Y, Reyburn H, Mbakilwa H, et al. The impact of response to the results of diagnostic tests for malaria: cost-benefit analysis. BMJ. 2008;336(7637):202–205. http://dx.doi.org/10.1136/bmj.39395.696065.47 18199700 10.1136/bmj.39395.696065.47 PMC2213875

[27] Hutchinson E, Reyburn H, Hamlyn E, et al. Bringing the state into the clinic? Incorporating the rapid diagnostic test for malaria into routine practice in Tanzanian primary healthcare facilities. Glob Public Health. 2015:1–15. [Epub ahead of print] http://dx.doi.org/10.1080/17441692.2015.1091025 10.1080/17441692.2015.1091025 PMC5526135 26457440

[28] Peeling RW, Mabey D Point-of-care tests for diagnosing infections in the developing world. Clin Microbiol Infect. 2010;16(8):1062–1069. http://dx.doi.org/10.1111/j.1469-0691.2010.03279.x 20670288 10.1111/j.1469-0691.2010.03279.x

[29] Almuhayawi M, Altun O, Stralin K, et al. Identification of microorganisms by FilmArray and matrix-assisted laser desorption ionization-time of flight mass spectrometry prior to positivity in the blood culture system. J Clin Microbiol. 2014;52(9):3230–3236. http://dx.doi.org/10.1128/JCM.01084-14 24951811 10.1128/JCM.01084-14 PMC4313143

[30] Babady NE The FilmArray^®^ respiratory panel: an automated, broadly multiplexed molecular test for the rapid and accurate detection of respiratory pathogens. Expert Rev Mol Diagn. 2013;13(8):779–788. http://dx.doi.org/10.1586/14737159.2013.848794 24151847 10.1586/14737159.2013.848794 PMC7103684

[31] Zheng X, Polanco W, Carter D, et al. Rapid identification of pathogens from pediatric blood cultures by use of the FilmArray blood culture identification panel. J Clin Microbiol. 2014;52(12):4368–4371. http://dx.doi.org/10.1128/JCM.02133-14 25274998 10.1128/JCM.02133-14 PMC4313318

[32] Assicot M, Bohuon C, Gendrel D, et al. High serum procalcitonin concentrations in patients with sepsis and infection. Lancet. 1993;341(8844):515–518. 8094770 10.1016/0140-6736(93)90277-N PMC7141580

[33] Tsalik EL, Henao R, Nichols M, et al. Host gene expression classifiers diagnose acute respiratory illness etiology. Science Translational Medicine. 2016;8(322):322ra11 http://dx.doi.org/10.1126/scitranslmed.aad6873 10.1126/scitranslmed.aad6873 PMC4905578 26791949

[34] Carrol ED, Mankhambo LA, Jeffers G, et al. The diagnostic and prognostic accuracy of five markers of serious bacterial infection in Malawian children with signs of severe infection. PLoS One. 2009;4(8):e6621 http://dx.doi.org/10.1371/journal.pone.0006621 19675669 10.1371/journal.pone.0006621 PMC2721152

[35] Bouckenooghe AR, Dupont HL, Jiang ZD, et al. Markers of enteric inflammation in enteroaggregative Escherichia coli diarrhea in travelers. Am J Trop Med Hyg. 2000;62(6):711–713. 11304060 10.4269/ajtmh.2000.62.711

[36] Huicho L, Campos M, Rivera J, et al. Fecal screening tests in the approach to acute infectious diarrhea: a scientific overview. Pediatr Infect Dis J. 1996;15(6):486–494. 8783344 10.1097/00006454-199606000-00004

[37] Okeke I The tragedy of antimicrobial resistance: achieving a recognition of necessity. Current Science. 2009;97(11):1564–1572.

[38] Bronzwaer SL, Cars O, Buchholz U, et al. A European study on the relationship between antimicrobial use and antimicrobial resistance. Emerg Infect Dis. 2002;8(3):278–282. http://dx.doi.org/10.3201/eid0803.010192 11927025 10.3201/eid0803.010192 PMC2732471

[39] Goossens H European status of resistance in nosocomial infections. Chemotherapy. 2005;51(4):177–181. http://dx.doi.org/10.1159/000086919 16006764 10.1159/000086919

[40] World Health Organization Antimicrobial resistance: global report on surveillance 2014 Geneva, Switzerland: WHO; 2014.

[41] Essack SY, Desta AT, Abotsi RE, et al. Antimicrobial resistance in the WHO African region: current status and roadmap for action. J Public Health. First published online: March 3, 2016 http://dx.doi.org/10.1093/pubmed/fdw015 10.1093/pubmed/fdw015 PMC5939661 26944074

[42] Klugman KP Efficacy of pneumococcal conjugate vaccines and their effect on carriage and antimicrobial resistance. Lancet Infect Dis. 2001;1(2):85–91. http://dx.doi.org/10.1016/S1473-3099(01)00063-9 11871480 10.1016/S1473-3099(01)00063-9

[43] Hanage WP, Finkelstein JA, Huang SS, et al. Evidence that pneumococcal serotype replacement in Massachusetts following conjugate vaccination is now complete. Epidemics. 2010;2(2):80–84. http://dx.doi.org/10.1016/j.epidem.2010.03.005 21031138 10.1016/j.epidem.2010.03.005 PMC2963072

[44] Okeke IN Cholera vaccine will reduce antibiotic use. Science. 2009;325(5941):674 http://dx.doi.org/10.1126/science.325_674b 10.1126/science.325_674b 19661401

[45] Waldor MK, Hotez PJ, Clemens JD A national cholera vaccine stockpile – a new humanitarian and diplomatic resource. N Engl J Med. 2010;363(24):2279–2282. http://dx.doi.org/10.1056/NEJMp1012300 21105832 10.1056/NEJMp1012300 PMC3970725

[46] Kiiru J, Mutreja A, Mohamed AA, et al. A study on the geophylogeny of clinical and environmental Vibrio cholerae in Kenya. PLoS One. 2013;8(9):e74829 http://dx.doi.org/10.1371/journal.pone.0074829 24066154 10.1371/journal.pone.0074829 PMC3774669

[47] Martin S, Costa A, Perea W Stockpiling oral cholera vaccine. Bull World Health Organ. 2012;90:714 http://dx.doi.org/10.2471/BLT.12.112433 23109735 10.2471/BLT.12.112433 PMC3471062

[48] Feasey NA, Houston A, Mukaka M, et al. A reduction in adult blood stream infection and case fatality at a large African hospital following antiretroviral therapy roll-out. PLoS One. 2014;9(3):e92226 http://dx.doi.org/10.1371/journal.pone.0092226 24643091 10.1371/journal.pone.0092226 PMC3958486

[49] Gandhi NR, Moll A, Sturm AW, et al. Extensively drug-resistant tuberculosis as a cause of death in patients co-infected with tuberculosis and HIV in a rural area of South Africa. Lancet. 2006;368(9547):1575–1580. http://dx.doi.org/10.1016/S0140-6736(06)69573-1 17084757 10.1016/S0140-6736(06)69573-1

[50] Labar AS, Millman JS, Ruebush E, et al. Regional dissemination of a trimethoprim-resistance gene cassette via a successful transposable element. PLoS One. 2012;7(5):e38142 http://dx.doi.org/10.1371/journal.pone.0038142 22666464 10.1371/journal.pone.0038142 PMC3364232

[51] Okoro CK, Kingsley RA, Connor TR, et al. Intracontinental spread of human invasive Salmonella typhimurium pathovariants in sub-Saharan Africa. Nat Genet. 2012;44(11):1215–1221. http://dx.doi.org/10.1038/ng.2423 23023330 10.1038/ng.2423 PMC3491877

[52] Cegelski L, Marshall GR, Eldridge GR, et al. The biology and future prospects of antivirulence therapies. Nat Rev Micro. 2008;6(1):17–27. http://dx.doi.org/10.1038/nrmicro1818 10.1038/nrmicro1818 PMC2211378 18079741

[53] Berkley JA, Lowe BS, Mwangi I, et al. Bacteremia among children admitted to a rural hospital in Kenya. N Engl J Med. 2005;352(1):39–47. http://dx.doi.org/10.1056/NEJMoa040275 15635111 10.1056/NEJMoa040275

[54] Opintan JA, Newman MJ, Nsiah-Poodoh OA, et al. Vibrio cholerae O1 from Accra, Ghana carrying a class 2 integron and the SXT element. J Antimicrob Chemother. 2008;62(5):929–933. http://dx.doi.org/10.1093/jac/dkn334 18755696 10.1093/jac/dkn334 PMC2566517

[55] Lamikanra A, Crowe JL, Lijek RS, et al. Rapid evolution of fluoroquinolone-resistant Escherichia coli in Nigeria is temporally associated with fluoroquinolone use. BMC Infect Dis. 2011;11:312 http://dx.doi.org/10.1186/1471-2334-11-312 22060770 10.1186/1471-2334-11-312 PMC3226678

[56] Rasko DA, Sperandio V Anti-virulence strategies to combat bacteria-mediated disease. Nat Rev Drug Discov. 2010;9(2):117–128. http://dx.doi.org/10.1038/nrd3013 20081869 10.1038/nrd3013

[57] Vale PF, McNally L, Doeschl-Wilson A, et al Beyond killing: can we find new ways to manage infection? EMPH. 2016;2016(1):148–157. http://dx.doi.org/10.1093/emph/eow012 27016341 10.1093/emph/eow012 PMC4834974

[58] Hardin G The tragedy of the commons. The population problem has no technical solution; it requires a fundamental extension in morality. Science. 1968;162(3859):1243–1248. 5699198

[59] Baquero F, Campos J The tragedy of the commons in antimicrobial chemotherapy. Rev Esp Quimioter. 2003;16(1):11–13. 12750754

[60] Eber MR, Laxminarayan R, Perencevich EN, et al. Clinical and economic outcomes attributable to health care-associated sepsis and pneumonia. Arch Intern Med. 2010;170(4):347–353. http://dx.doi.org/10.1001/archinternmed.2009.509 20177037 10.1001/archinternmed.2009.509

[61] Klein E, Smith DL, Laxminarayan R Hospitalizations and deaths caused by methicillin-resistant Staphylococcus aureus, United States, 1999–2005. Emerg Infect Dis. 2007;13(12):1840–1846. http://dx.doi.org/10.3201/eid1312.070629 18258033 10.3201/eid1312.070629 PMC2876761

[62] Aboderin AO, Adefehinti O, Odetoyin BW, et al. Prolonged febrile illness due to CTX-M-15 extended-spectrum β-lactamase-producing Klebsiella pneumoniae infection in Nigeria. Afr J Lab Med. 2012;1(1), Art. #16, 4 pages. http://dx.doi.org/10.4102/ajlm.v1i1.16 10.4102/ajlm.v1i1.16 PMC5644516 29062733

[63] Laxminarayan R, Matsoso P, Pant S, et al. Access to effective antimicrobials: a worldwide challenge. Lancet. 2016;387(10014):168–175. http://dx.doi.org/10.1016/S0140-6736(15)00474-2 26603918 10.1016/S0140-6736(15)00474-2

[64] Laxminarayan R, Amábile-Cuevas CF, Cars O, et al UN high-level meeting on antimicrobials—what do we need? Lancet. 2016;388(10041):218-220. http://dx.doi.org/10.1016/S0140-6736(16)31079-0 27479554 10.1016/S0140-6736(16)31079-0

[65] O’Neill J Review on antimicrobial resistance: Antimicrobial resistance: Tackling a crisis for the health and wealth of nations. London, UK: Wellcome Trust; 2014.

[66] Frean J, Perovic O, Fensham V, et al External quality assessment of national public health laboratories in Africa, 2002-2009. Bull World Health Organ. 2012;90(3):191–199A. http://dx.doi.org/10.2471/BLT.11.091876 22461714 10.2471/BLT.11.091876 PMC3314205

[67] Gandra S, Merchant AT, Laxminarayan R A role for private sector laboratories in public health surveillance of antimicrobial resistance. Future Microbiol. 2016;11:709–712. http://dx.doi.org/10.2217/fmb.16.17 27192102 10.2217/fmb.16.17

[68] World Health Organization Global antimicrobial resistance surveillance system: Manual for early implementation. Geneva, Switzerland: WHO; 2015.

[69] Govender NP, Patel J, Magobo RE, et al. Emergence of azole-resistant Candida parapsilosis causing bloodstream infection: results from laboratory-based sentinel surveillance in South Africa. J Antimicrob Chemother. 2016;71(7):1994–2004. http://dx.doi.org/10.1093/jac/dkw091 27125552 10.1093/jac/dkw091 PMC11911919

[70] Köser CU, Ellington MJ, Cartwright EJ, et al. Routine use of microbial whole genome sequencing in diagnostic and public health microbiology. PLoS Pathogens. 2012;8(8):e1002824 http://dx.doi.org/10.1371/journal.ppat.1002824 22876174 10.1371/journal.ppat.1002824 PMC3410874

[71] Rubach MP, Hanson KE ID Learning Unit – Diagnostics update: Current laboratory methods for rapid pathogen identification in patients with bloodstream infections. Open Forum Infect Dis. 2015;2(4):ofv174 http://dx.doi.org/10.1093/ofid/ofv174 26719845 10.1093/ofid/ofv174 PMC4690501

